# Mucosal-Associated Invariant T Cells are not susceptible *in vitro* to SARS-CoV-2 infection but accumulate into the lungs of COVID-19 patients

**DOI:** 10.1016/j.virusres.2024.199315

**Published:** 2024-01-13

**Authors:** Xiaobo Huang, Jonas Kantonen, Kirsten Nowlan, Ngoc Anh Nguyen, Suvi T. Jokiranta, Suvi Kuivanen, Nelli Heikkilä, Shamita Mahzabin, Anu Kantele, Olli Vapalahti, Liisa Myllykangas, Santtu Heinonen, Mikko I. Mäyränpää, Tomas Strandin, Eliisa Kekäläinen

**Affiliations:** a Translational Immunology Research Program, University of Helsinki and Helsinki University Hospital, Helsinki, Finland; b Department of Bacteriology and Immunology, University of Helsinki, Helsinki, Finland; c Department of Pathology, University of Helsinki, Helsinki, Finland; d Department of Pathology, HUS Diagnostic Center, Helsinki University Hospital, Helsinki, Finland; e Department of Virology, Medicum, University of Helsinki, Helsinki, Finland; f Charité-Universitätsmedizin Berlin, Corporate Member of Freie Universität Berlin, Humboldt-Universität zu Berlin, and Berlin Institute of Health, Institute of Virology, Berlin, Germany; g Center of Vaccinology, University of Geneva, Geneva, Switzerland; h Human Microbiome Research Program, Faculty of Medicine, University of Helsinki, Helsinki, Finland; i Meilahti Vaccine Research Center, MeVac, Infectious Diseases, Helsinki University and Helsinki University Hospital, Helsinki, Finland; j Division of Virology and Immunology, HUS Diagnostic Center, HUSLAB Clinical Microbiology, Helsinki, Finland; k Zoonosis Unit, Department of Virology, Medicum, University of Helsinki, Helsinki, Finland; l Department of Veterinary Biosciences, University of Helsinki, Helsinki, Finland; m New Children's Hospital, Pediatric Research Center, University of Helsinki and Helsinki University Hospital, Helsinki, Finland

**Keywords:** Mucosal Associated Invariant T cells, SARS-CoV-2, Lymphopenia, COVID-19, Viral challenge

## Abstract

Prolonged T cell lymphopenia is common in COVID-19, caused by SARS-CoV-2. While the mechanisms of lymphopenia during COVID-19 remain elusive, it is especially pronounced in a specialized innate-like T cell population called Mucosal Associated Invariant T cells (MAITs). MAITs has been suggested to express Angiotensin-Converting Enzyme 2 (ACE2), which is the well-known cellular receptor for SARS-CoV-2. However, it is still unclear if SARS-CoV-2 can infect or affect MAIT cells directly. In this study, we performed multicolor flow cytometry on peripheral blood mononuclear cells obtained from COVID-19 patients to assess the frequencies of CD8^+^Vα7.2^+^CD161^+^ MAIT subsets at acute and convalescent disease phases. The susceptibility of MAITs and T cells to direct exposure by SARS-CoV-2 was analysed using cells isolated from healthy donor buffy coats by viability assays, virus-specific RT-PCR, and flow cytometry. *In situ* lung immunofluorescence was used to evaluate retention of T cells, especially MAIT cells, in lung tissues during acute COVID-19. Our study confirms previous reports indicating that circulating MAITs are activated, and their frequency is declined in patients with acute SARS-CoV-2 infection, whereas an accumulation of MAITs and T cells was seen in the lung tissue of individuals with fatal COVID-19. However, despite a fraction of MAITs found to express ACE2, no evidence for the susceptibility of MAITs for direct infection or activation by SARS-CoV-2 particles was observed. Thus, their activation and decline in the circulation is most likely explained by indirect mechanisms involving other immune cells and cytokine-induced pro-inflammatory environment but not by direct exposure to viral particles at the infection site.

## Highlights

• Circulating CD8^+^CD161^+^Vα7.2^+^ MAITs were highly proliferative and activated, but their numbers were decreased during acute SARS-CoV-2 infection.
• Direct exposure to SARS-CoV-2 viral particles did not infect or activate MAIT cells.
• T cells and CD3^+^Vα7.2^+^ cells, that are enriched for MAIT cells, accumulated in the lungs of the fatal COVID-19 cases studied.

## 1 Introduction

The spread of Severe Acute Respiratory Syndrome Coronavirus 2 (SARS-CoV-2) has led to an ongoing worldwide pandemic of coronavirus disease 2019 (COVID-19). Lymphopenia is a common finding in severe and moderate cases of COVID-19 (Tan et al., 2020) and its magnitude can predict disease severity (Huang and Pranata, 2020). However, the mechanisms leading to lymphopenia during COVID-19 are still to a large extent unclear. Lymphopenia is more pronounced in a specific T cell population called Mucosal Associated Invariant T (MAIT) cells that are innate-like T cells enriched on mucosal surfaces (Jouan et al., 2020a; Parrot et al., 2020; Flament et al., 2021). In patients with COVID-19, decline of circulating MAIT cell frequencies and the presence of highly activated MAIT cells are reported by several studies (Deschler et al., 2021; Flament et al., 2021; Parrot et al., 2020; Youngs et al., 2021; Yu et al., 2021) while evidence is still lacking whether MAIT activation has a beneficial or pathogenic role in the infection.

MAIT cells are a relatively recently identified population of innate-like T cells, which represent 1–5 % of T cells in the circulation in adults and have strong tissue homing characteristics especially to liver and lung (Provine and Klenerman, 2020). MAITs are defined based on the expression of their invariant T cell receptor and many innate receptors, such as CD161, which are mostly expressed by innate lymphocytes like natural killer cells (Provine and Klenerman, 2020). MAITs recognize and are activated by bacterial and fungal metabolites presented in an evolutionary conserved non-variable major histocompatibility complex-like molecule MR1 (MHC-related 1) that is expressed on many different cell types (Godfrey et al., 2019). Once activated, MAITs display immediate effector functions by secreting inflammatory cytokines and cytotoxic molecules, which can kill infected cells (Le Bourhis et al., 2013). Therefore, they are believed to have a significant role in mucosal early responses to bacterial and fungal pathogens. MAIT cells can be also activated in a non-specific manner through cytokines, which suggests that MAITs could also play a role in the immune defence of viral infections (Ussher et al., 2014). However, their exact role in the pathogenesis or protection in viral diseases is still unknown (Loh et al., 2016; Ussher et al., 2018; van Wilgenburg et al., 2016). In addition to their pro-inflammatory and cytotoxic functions, MAITs can also have a homeostatic role through production of IL-22 that mediates wound and epithelial healing (Leeansyah et al., 2014).

T cells generally do not express the SARS-CoV-2 receptor Angiotensin-Converting Enzyme 2 (ACE2) and productive or cytopathic infection of T lymphocytes with the virus has not been convincingly shown (Kuklina, 2022). Interestingly, MAITs have been suggested to express low levels of the ACE2 receptor on mRNA level (Liu et al., 2020). This does not necessarily translate to ACE2 protein expression, which to our knowledge has not been directly addressed on MAITs. It is therefore possible that SARS-CoV-2 has a direct effect on MAIT cells through receptor binding and even infection.

In the current study, we analyzed the frequencies and activation status of circulating MAITs in patients with acute and convalescent stage of COVID-19 infection and with variable disease severities. The extravasation of MAITs into lung tissue was analyzed *in situ* for fatal COVID-19 cases. To assess the potential infectivity and/or activation of MAITs by SARS-CoV-2, we challenged MAITs isolated from healthy controls with purified virus. Our results confirm and expand previous reports on the important role of MAITs during and after SARS-CoV-2 infection.

## 2 Materials and methods

### 2.1 Patients and healthy donors

SARS-CoV-2 infected patients aged from 27 to 77 years old (*n* = 40) with acute COVID-19 (confirmed by RT-PCR) admitted to the Helsinki University Hospital, Helsinki, Finland, or in convalescent disease phases, were recruited to this study. Blood samples were collected at different time points after symptom onset and grouped as follows: Samples taken less than 21 days after symptom onset were defined as acute (*n* = 18), from 21 to 90 days as early convalescent (*n* = 21) and over 90 days as late convalescent (*n* = 23). One sample from an individual patient was included in each disease phase group, none of the patients had samples in all three disease phases. Plasma samples were collected together with the blood samples and frozen immediately after processing. Lung tissue (*n* = 3, age range 41–77 years, 1 male, 2 females) were collected at autopsy for *in situ* immunofluorescence (IF) staining from patients who died from a RT-PCR confirmed SARS-CoV-2 infection. Anonymous lung tissue samples obtained from lung resections done for non-infectious reasons (*n* = 5) were used as controls. Healthy anonymous blood donor buffy coats (*n* = 9) for Vα7.2^+^ cell enrichment were obtained from Finnish Red Cross blood service, Helsinki, Finland. The COVID-19 patients’ demographic information is summarized in Table 1.

**Table 1:** Clinical characteristics of COVID-19 patients and healthy donors.

Characteristic		All patients	Acute sample group	Early convalescent sample group	Late convalescent sample group
n (%)		40 (100)	18 (48)	21 (53)	23 (58)
Treatment condition (%)	ICU	11 (28)	5 (28)	4 (19)	8 (35)
	Hospitaliza-tion	15 (38)	13 (72)	3 (14)	8 (35)
	Home	14 (35)	–	14 (67)	7 (30)
Age, years (median)		55.0	54.0	–	–
Age, years IQR	25th	36.0	–	–	–
	75th	61.3	–	–	–
Female (%)		24 (56)	23 (58)	–	–
Male (%)		19 (44)	17 (42)	–	–
DSO (median)		–	10	27	136
DSO range		–	2 - 20	21 - 81	96 - 417

IQR: Interquartile range; DSO: Days from symptom onset before sampling; 2 samples overlapped in acute and early convalescent groups; 11 samples overlapped in acute and late convalescent groups; 10 samples overlapped in early convalescent and late convalescent groups.

The study was approved by the Ethics Committee of the Hospital District of Helsinki and Uusimaa (HUS/853/2020, HUS/1238/2020). All living patients gave a written informed consent in accordance with the Declaration of Helsinki. Autopsies were performed in accordance with Finnish legislation, with permission granted by the next of kin of the deceased.

### 2.2 Cell isolation and enrichment

Peripheral blood mononuclear cells (PBMCs) from COVID-19 patients and healthy donor buffy coats were isolated by density gradient centrifugation using Cytiva Ficoll-Paque PLUS Media (Thermo Fisher). Isolated PBMCs from COVID-19 patients were frozen with CTL kit (cat: CTLC-ABC, ImmunoSpot) and then stored in liquid nitrogen until use. T cells were isolated from PBMCs of healthy donor buffy coats by using the Pan T Cell Isolation Kit (Miltenyi Biotec). Vα7.2^+^ cells from PBMCs of healthy donor buffy coats were enriched via positive selection with Anti-PE MicroBeads UltraPure (Miltenyi Biotec) after staining with PE-labeled Vα7.2 monoclonal antibody (clone: 3C10, Biolegend).

The purity of T cells and classical MAIT-cell contents of the Vα7.2^+^ enriched cells used in the *in vitro* viral challenges was assessed by CD3 or 5-OP-RU loaded MR1 tetramer staining (NIH Tetramer Core Facility, Emory University, Atlanta, GA, The USA) (Corbett et al., 2014) and flow cytometry analysis. 31 % of the Vα7.2^+^ enriched cells were MR1-tetramer positive classical MAITs (Supplementary Fig. 1A) and > 90 % of the magnetic bead isolated T cells were CD3-positive (Supplementary Fig. 1B).

### 2.3 *In vitro* viral challenge

Vero E6 cells (green monkey kidney epithelial cell line; ATCC no. CRL-1586) were grown in Minimum Essential Medium (MEM; Sigma-Aldrich, St. Louis, MO, USA). Wild type SARS-CoV-2 (Wuhan strain; CIP1) (Cantuti-Castelvetri et al., 2020) was grown on Vero E6 cells expressing TMPRSS2 (Rusanen et al., 2021) and purified from cell culture supernatant by ultracentrifugation through 30 % sucrose cushion (SW28 rotor; 28,000 rpm, 90 min, 4 °C). Virus titers were calculated by the median tissue culture infectious dose (TCID50) after assessing cytopathic effects by crystal violet staining of cell cultures infected for 5 days with serially diluted virus.  PBMCs, enriched T cells and MAITs were challenged with multiplicity of infection (MOI) of 0.5, 1 or 10 in R10 medium (RPMI-1640 supplemented with 10 % inactivated FCS, 100 IU/mL of Penicillin and 100 µg/mL of Streptomycin and 2 mM of L-glutamine) for 1 h, 24 h or 72 h at 37 °C, 5 % CO_2_. Vero E6 cells were used as positive control and cells without viral challenge (Mock) or challenged with UV-inactivated virus (UV; 300,000 μJ/cm2, Stratalinker, Stratagene) were used as negative control. All studies with SARS-CoV-2 were performed in a Biosafety level 3 laboratory (BSL3).

### 2.4 Flow cytometry

The antibodies used for staining are summarised in Supplementary Table 1. LIVE/DEAD fixable Green Dead Cell Stain Kit (Thermo Fisher Scientific) and LIVE/DEAD Fixable Aqua Dead Cell Stain Kit (Thermo Fisher Scientific) were used for staining of live/dead cells. For testing ACE2 expression on lymphocytes, PBMCs from healthy donors were stained separately with ACE2 antibody only, Goat IgG antibody (Alexa Fluor 488) only, and both ACE2 and Goat IgG antibody and, together with unstained PBMCs as Fluorescence Minus One Controls (FMOs). PBMCs from 3 healthy donors were stained with LIVE/DEAD Fixable Aqua Dead Cell Stain Kit (Thermo Fisher Scientific), ACE2 antibody, Goat IgG antibody (Alexa Fluor 488), together with CD3-BV786 and MR1/5-OP-RU-APC (Supplementary Fig. 2). For the COVID-19 patient samples, cells were first quickly thawed in a 37 °C water bath, then washed with CTL wash (ImmunoSpot). After washing, cells were stained with surface markers and live/dead cell markers for 30 min at 4 °C in Horizon Brilliant Staining Buffer (BD). After washing, cells were fixed and permeabilized in Foxp3/Transcription Factor Fixation/Permeabilization Concentrate and Diluent (cat: 00-5521-00, eBioscience) for 40 min at 4 °C and stained with intracellular markers in 1X Permeabilization Buffer (cat: 00-8333-56, eBioscience) for 30 min at 4 °C, then washed. For viral challenged samples in BSL3, cells were first stained with surface markers and live/dead cell markers for 20 min room temperature (RT), then washed with PBS + 0.2 % BSA. After washing, cells were incubated with 4 % paraformaldehyde for 15 min at RT. After washing, cells were suspended in PBS with 0.1 % Triton-X. Samples were acquired on a BD Fortessa LSRII flow cytometry (BD Biosciences, USA) and then analysed with Flowjo v10.7.2.

### 2.5 Cell viability assay

At the end of 24 h or 72 h virus challenge, the viability of MAITs was measured with a kit designed to quantify ATP level according to instructions of the manufacturer (CellTiterGlo 2.0 Luminescent Cell Viability Assay, Promega) using Hidex Sense microplate reader (Hidex Oy, Finland).

### 2.6 RNA extraction and real-time qPCR

Total RNA was extracted from cells challenged with the virus using the Qiagen RNeasy Minikit (Qiagen) according to the manufacturer's guidelines. The amount of virus was quantified by one–step reverse–transcriptase quantitative polymerase chain reaction (RT–qPCR) with fluorescent primer–probe sets employing 1–step fast virus master mix (cat: 4444434, Thermo Scientific; Breda, The Netherlands) and AriaMx real–time PCR instrumentation (Agilent, Santa Clara, CA, USA). The virus was detected using primers and probe for viral RNA dependent RNA polymerase (RdRp): forward 5′-gtgaratggtcatgtgtggcgg-3′, reverse 5′-caratgttaaasacactattagcata-3′ and probe FAM-5′- caggtggaacctcatcaggagatgc-3′ (Corman et al., 2020). Samples were normalized based Glyceraldehyde-3-Phosphate Dehydrogenase (GAPDH) mRNA expression as assessed using a commercial VIC-fluorochrome based primer-probe set (cat: 4326317E, Applied Biosystems™). The relative expression of RdRp in comparison to UV-inactivated virus challenge was calculated by the 2^–deltadeltaCt method (Corbett et al., 2014) which is 2^ (deltaCt of UV–deltaCT of virus). The RT-PCR cycling conditions were 48 °C 10 min, 95 °C 3 min and 95 °C 5 s, 55 °C 30 s, 68 °C 20 s in 40 cycles.

### 2.7 Interleukin 18 ELISA

Plasma samples were analysed for Interleukin 18 (IL-18) concentration in duplicates with Human Total IL-18 DuoSet ELISA kit per manufacturer's instructions (Bio-Techne, Minneapolis, the USA) and 450 nm absorbance values were measured with a Byonoy microplate reader (Hamburg, Germany).

### 2.8 Lung tissue immunofluorescence staining

6 μm thick frozen sections of lung tissue were air-dried for 30 min at RT. Sections were subsequently fixed using cold acetone-methanol (1:1) for 10 min and washed (PBS at RT, 3 × 10 min). Next, sections were treated with Odyssey Blocking Buffer for 60 min at RT. A primary mixture of antibodies Rat-anti-Human CD3; Mouse-anti-Human TCR Vα7.2 and Hoechst stain was prepared with Odyssey Blocking Buffer to get concentrations of 3.3 μg/ml for antibodies and 1 μg/ml for the Hoechst stain, respectively. Sections were stained using the primary mixture overnight at +4 °C and washed (PBS at RT, 3 × 10 min). Secondary staining was performed in three steps. First, sections were stained with HRP-labelled Rabbit-anti-Mouse antibody at 1:6 concentration in Odyssey Blocking Buffer for 30 min at RT and washed (PBS at RT, 3 × 10 min). Second, sections were incubated with 1:2000 Cy5-reagent in TSA-buffer for 10 min at RT and washed (PBS at RT, 3 × 10 min). Third, sections were stained with Alexa Fluor 488-labelled Donkey-anti-Rat antibody at 1:1000 concentration in Odyssey Blocking Buffer and washed (PBS at RT, 3 × 10 min). Finally, sections were covered using Prolong Gold mountant (Thermo Fisher). The slides were scanned as whole-slide images (WSIs) with 3DHISTECH Pannoramic 250 FLASH II digital slide scanner at the Genome Biology Unit supported by HiLIFE and the Faculty of Medicine, University of Helsinki, and Biocenter Finland.

The visible streaks seen in the images acquired with the green channel are caused by autofluorescence of elastic fibers. The autofluorescent spectrum of elastin and collagen is broad (Favreau et al., 2020) and hinders obtaining highly specific images by any fluorophore in lung tissue immunofluorescence microscopy, as is evident from lung sections without any primary antibody staining (Supplementary Fig. 3).

### 2.9 Annotation of lung tissue whole slide images and tissue cell counts

WSIs were assessed using the CaseViewer software (3DHISTECH, Version 2.4) by two pathologists to quantify the accumulation of CD3^+^ and CD3^+^Vα7.2^+^ cells. MAIT cells represent a significant subset of the CD3^+^Vα7.2^+^ cells, and CD3^+^Vα7.2^+^ counts were used as surrogate for MAIT counts. Given the significant autofluorescence of fibers such as elastin and collagen in the lung tissue, true positive cellular staining compatible with MAIT cells was determined using morphological criteria (see below) to exclude signal generated by autofluorescence. Five regions of interest (ROI) were selected representing the areas of highest apparent cell counts within each slide, with the ROI annotated using the software's “Draw a 40 x FOV sized circle” function, resulting in a total area of 1.5 mm² assessed for each slide. CD3^+^ cells showing a nucleus, lymphocyte morphology and plasma membrane CD3^+^ staining were annotated first within the drawn ROIs using the software's “Draw an arrow” function, whereafter the cells showing plasma membrane Vα7.2^+^ staining amongst the annotated CD3^+^ cells were further annotated using the software's “Draw a fixed size circle” function. The annotations for each slide were then exported as a csv-file and cell counts were generated by counting the numbers of annotations in Excel for Microsoft 365 software (Microsoft corporation, Office 16).

### 2.10 Statistical analysis

Statistical analysis was performed using Graphpad Prism software version 9 and R studio (v4.0.2). Kruskal-Wallis one-way ANOVA test was used when comparing multiple groups and Mann-Whitney U-test was used when comparing between two unpaired groups. Wilcoxon signed rank test or paired T-test were used with paired group comparison, depending on the sample size (T-test if sample size was less than 6). Values are expressed as mean ± standard deviation (SD). Statistical significance is recognized at *P* < 0.05. *P < 0.05, **P < 0.01, ***P < 0.001, ns means no statistical significance. Correlations were calculated between the frequencies of CD8^+^CD161^+^Vα7.2^+^MAITs of CD8^+^ cells, IL-18 concentration, and patients' clinical data (age, gender and treatment) using the Spearman correlation with ‘ggcorrplot’ R package.

## 3 Results

### 3.1 MAIT cells are activated and their frequency declines in acute COVID-19

To assess the frequency and function of circulating MAIT cells during COVID-19, we collected 62 prospective samples from 40 individuals that had COVID-19 due to PCR-confirmed SARS-CoV-2. Samples were grouped as acute (*n* = 18), early convalescent (*n* = 21), and late convalescent (*n* = 23) and analyzed with a multi-color cytometry. Clinical characteristics of the patients are summarized in Table 1. It has been reported that in COVID-19 patients the MAIT cell identification as CD8^+^CD161^+^Vα7.2^+^highly overlaps with the identification done with the MR1/5-OP-RU tetramer, which is considered the most specific method to identify MAITs (Flament et al., 2021). In a subset of our COVID-19 patient samples (*n* = 38), we were able to confirm that 80 % (median, range 36–93 %, Supplementary Fig. 1C) of the CD3^+^CD8^+^CD161^+^Vα7.2^+^ cells were also positive for the MR1-tetramer. Therefore, we decided to use CD8^+^CD161^+^Vα7.2^+^ as our definition of MAITs (gating strategy for MAITs is shown in Supplementary Fig. 4).

**Fig. 1:**
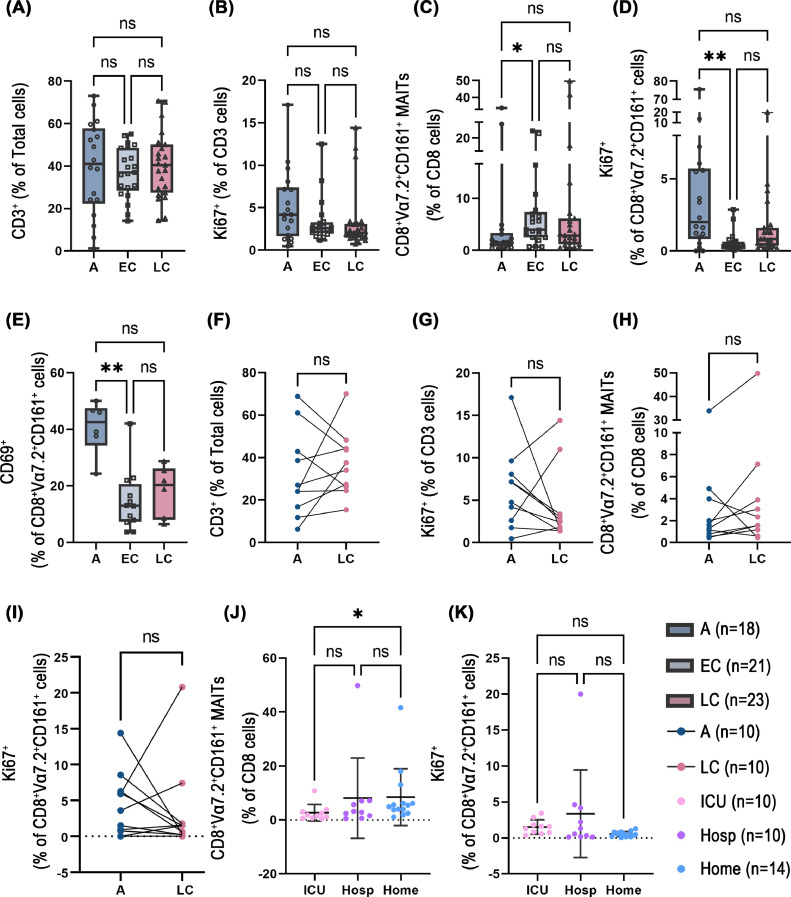
Frequencies of circulating MAITs during COVID-19. Unpaired (A–E) and paired (matched) (F–I) patient sample analysis in acute and convalescent phases. (A) and (F) The proportion of CD3^+^ cells of the total number of PBMCs. (B) and (G) The proportion of Ki67^+^ cells of total CD3^+^ cells. (C), (H) and (J) The proportion of CD8^+^Vα7.2^+^CD161^+^ MAITs of CD8^+^ cells. (D), (I) and (K) The proportion of Ki67^+^ cells of CD8^+^Vα7.2^+^CD161^+^ MAITs. (E) The proportion of CD69^+^ cells of CD8^+^Vα7.2^+^CD161^+^ MAITs. (J) and (K) Unpaired convalescent patient samples analysis among ICU-, hospitalized non-ICU and home-treated patients. Of note, CD69 was only analyzed in a subset of samples (Acute: *n* = 6, Early convalescent: *n* = 12, Late convalescent: *n* = 6). A, Acute. EC, Early convalescent. LC, Late convalescent. ICU, ICU-treated patients. Hosp, Hospitalized non-ICU patients. Home, Home-treated patients. Data were shown as mean ± SD. Kruskal-Wallis one-way ANOVA, Wilcoxon and Mann-Whitney U tests were used to test for statistical significance. *P < 0.05, **P < 0.01, ns = no statistical significance.

In our cohort, we could not detect a significant general T cell lymphopenia (Fig. 1A) or T cell activation in acute samples compared to convalescence as measured by the expression of a proliferation marker Ki67 on CD3^+^ cells (Fig. 1B). Also the proportion of total CD3^+^ cells out of the total number of PBMCs or Ki67^+^ proliferating T cells was unchanged in paired samples comparison of acute and convalescent samples from the same donor (Fig 1F, G). However, the proportion of CD8^+^CD161^+^Vα7.2^+^ MAITs out of all CD8^+^ cells was significantly lower in the acute samples as compared with the early convalescent samples (Fig. 1C). A trend towards a lower proportion of MAITs was seen in the acute samples as compared with the late convalescent samples in the paired analysis (Fig. 1H) but the difference was statistically insignificant, probably due to the low number of paired samples obtained from the same individuals. A significantly higher proportion of CD8^+^CD161^+^Vα7.2^+^ MAITs expressed proliferation marker Ki67 and activation marker CD69 in the acute samples indicating active proliferation and activation during acute COVID-19 as compared to convalescence (Fig. 1D, E).

We compared the frequency of the CD8^+^CD161^+^Vα7.2^+^ MAITs out of all CD8^+^ T cells during acute disease in patients who were treated in the intensive care unit (ICU) with patients that were hospitalized without the need of ICU. The frequencies of proliferating or activated CD8^+^CD161^+^Vα7.2^+^ MAITs, based on the expression of Ki67 or CD69 respectively, were not different in ICU treated patients compared to non-ICU patients (Supplementary Fig. 5). Due to self-isolation precautions, we could not obtain samples from acute COVID-19 outpatients. However, when we compared the frequency of CD8^+^CD161^+^Vα7.2^+^ MAITs out of all CD8^+^ T cells during convalescence in patients who were treated in the ICU, hospital but non-ICU or at home, the frequencies of MAITs were significantly lower in ICU treated patients compared to other groups. (Fig. 1J). However, we found no significant difference in the proliferating CD8^+^CD161^+^Vα7.2^+^ MAITs in convalescent samples (Fig. 1K). The clinical patient data is summarized in Supplementary Table 2. These data are well in line with previous reports indicating activation and decreased frequencies of MAITs in circulation of acute COVID-19 (Deschler et al., 2021; Flament et al., 2021; Parrot et al., 2020; Youngs et al., 2021; Yu et al., 2021; Yang et al., 2021).

Altogether, these results show that peripheral MAIT cells are proportionally decreased and activated in acute COVID-19. However, in the ICU treated patients, the proportion of CD8^+^CD161^+^Vα7.2^+^ MAITs remained low also during convalescence.

### 3.2 T cells and MAIT cells survive after *in vitro* viral challenge

**Fig. 2:**
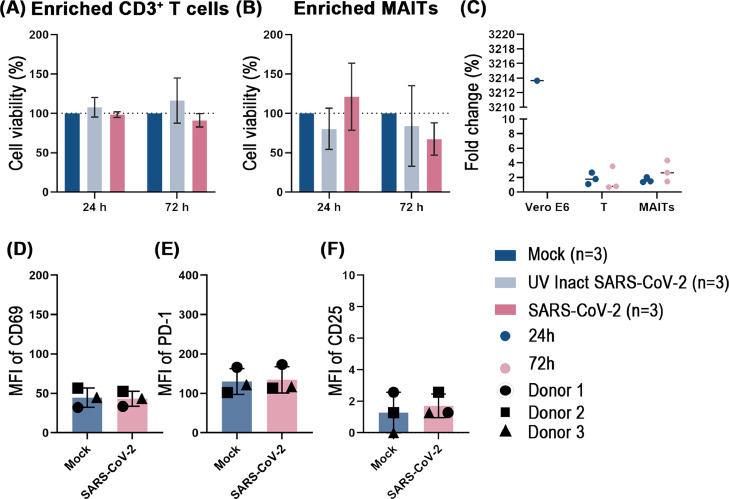
The viability and viral replication in T and MAIT cells, and Median Fluorescence Intensity (MFI) of MAITs functional markers after *in vitro* challenge with SARS-CoV-2. The viability of T cells (A) and MAIT cells (B) were measured by assessing a released ATP after 24 h and 72 h, respectively, after *in vitro* viral challenge. The Mock-infected samples were set as 100 % viable (dotted lines). The percentage viability of UV-inactivated SARS-CoV-2 and SARS-CoV-2 challenged T and MAIT cells were calculated based on the Mock-infected group. Viral replication on T cells and MAIT cells (C) were determined by the relative expression of viral Rdrp RNA in comparison to cells challenged with UV-inactivated virus (fold change) after 24 h and 72 h of *in vitro* viral challenge. Vero E6 cells infected with SARS-CoV-2 for 24 h served as a positive control. MFI of CD69 (D), PD-1 (E) and CD25 (F) on CD8^+^ MAIT cells after 24 h of *in vitro* viral challenge. Kruskal-Wallis one-way ANOVA and Mann-Whitney U test were used to test for statistical significance. No statistical significance was observed.

Productive infection of PBMCs with SARS-CoV-2 *in vitro* has been reported which could explain the loss of lymphocytes through increased apoptosis (Pontelli et al., 2022). We therefore challenged unfractionated PBMCs from healthy non-vaccinated SARS-CoV-2 seronegative blood donors with SARS-CoV-2 (0.5 MOI). In line with the published results, we could detect a 16- and 37-fold increase in the viral RNA during the 72 h challenge with RT-PCR (data not shown). These data motivated us to challenge enriched MAITs, because a small fraction of circulating MAITs appear to express the SARS-CoV-2 receptor ACE2 (Supplementary Fig. 2). Thus, we investigated whether the viability of enriched Vα7.2^+^ cells (and CD3^+^ T cells as reference) isolated from healthy individuals is affected when being exposed to live SARS-CoV-2 *in vitro*. Cell viability, as measured by released cellular ATP after virus exposure, showed comparable levels between purified live SARS-CoV-2 virus challenged cells (MOI 10) and untreated mock cells or cells challenged with UV-inactivated SARS-CoV-2 virus (Fig. 2A and B), indicating that the cell viability of MAITs or T cells is not significantly affected after being exposed to SARS-CoV-2 for 24 h and 72 h. We also investigated whether the viability of enriched Vα7.2^+^ cells is affected when being exposed to lower MOI (MOI 1) of live SARS-CoV-2 *in vitro* for 48 h with similarly negative results (Supplementary Fig. 6).

### 3.3 T cells and MAIT cells are not directly infected or activated by SARS-CoV-2

We further determined whether SARS-CoV-2 can directly infect T cells and specifically the ACE2 expressing MAIT cells. To assess viral replication in infected cells, we quantified the expression of viral RNA-dependent RNA polymerase (RdRp) RNA at 24 h and 72 h after exposure to live SARS-CoV-2 virus (MOI 10) in comparison to cells exposed to the same amount of UV-inactivated SARS-CoV-2, by RT-qPCR (Favaro et al., 2021). The results showed no significant differences in the level of RdRp RNA in cells exposed to live or UV-inactivated SARS-CoV-2 (Fig. 2C), indicating no significant replication of SARS-CoV-2 in MAITs. Not surprisingly, similar results were found also in the case of CD3^+^ T cells while SARS-CoV-2 replicated efficiently in Vero E6 cells, serving as the positive control. In conclusion, T cells or MAIT cells are not susceptible to infection by SARS-CoV-2.

We next investigated the expression of immune activation and exhaustion markers, CD69, CD25 and PD-1, in MAIT cells after being exposed to purified live SARS-CoV-2 virus (Fig. 2D–F). We found no significant change in the expression of CD69, CD25 and PD-1 on CD8^+^ MAIT cells after exposure to live virus (MOI 10) or untreated mock cells. In summary, these data suggests that MAIT cells are not activated even when being directly exposed to high concentration of purified live SARS-CoV-2.

### 3.4 T cells and MAIT cells accumulate in the lung tissue of COVID-19 patients

We next examined possible other causes for the MAIT cell lymphopenia. Excessive amounts of proinflammatory cytokines during acute COVID-19 have been suggested to cause MAIT cell overactivation which also could lead to increased apoptosis (Flament et al., 2021). We therefore measured IL-18 concentrations in a subset of our patients (patients *n* = 11, acute samples *n* = 8, late convalescent *n* = 9). As expected, the IL-18 concentration in the acute disease phase was significantly higher than in the late convalescent phase both with unpaired and paired samples (Supplementary Fig. 7A and B). However, we could not detect a significant correlation between IL-18 concentration and proportion of MAIT cells in the acute samples.

**Fig. 3:**
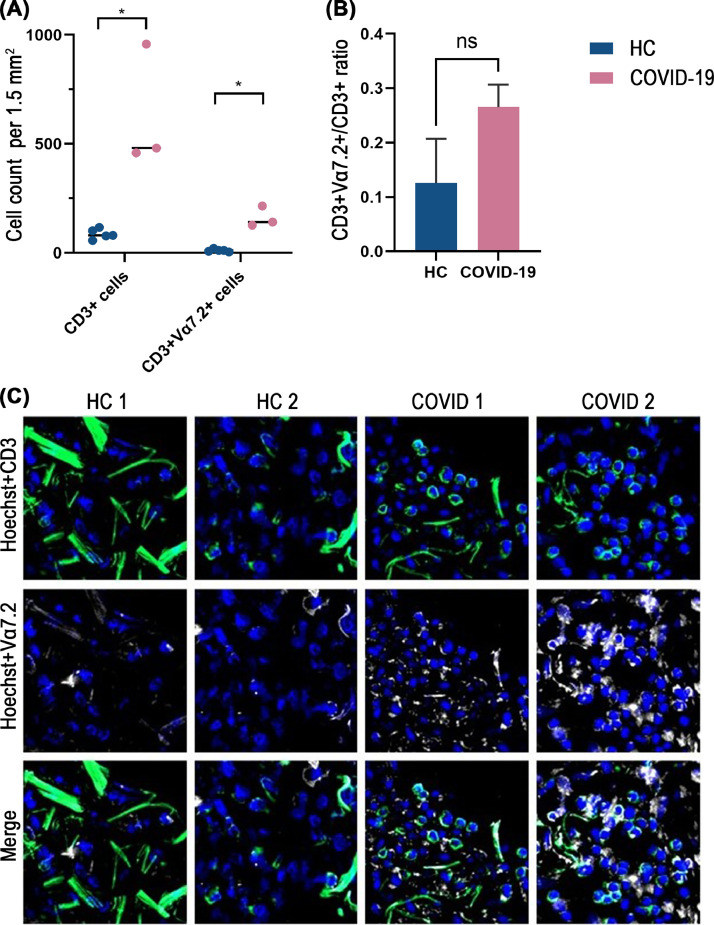
Lung infiltration of T cells and MAITs in COVID-19. Lung tissue immunofluorescence analysis (pseudo-color images) of CD3^+^ cells and CD3^+^Vα7.2^+^ cells stained in lung tissue sections from COVID-19 decedents and healthy controls (HC). Nuclear stain (Hoechst) is shown blue, CD3 shown green and Vα7.2 shown white. Streaks of green fibers are autofluorescent structures of the lung alveolar walls, consisting mainly of elastin and collagen. (A) CD3^+^and CD3^+^Vα7.2^+^ cell counts in HC and COVID-19 cases per 1.5 mm^2^. (B) Normalized CD3^+^Vα7.2^+^ cell counts in HC and COVID-19 cases. (C) Panel of images representative of HC and COVID-19 lung tissue sections at 200 x magnification (CaseViewer software). Mann-Whitney U test was used to test for statistical significance. Data shown as means ± SD. *P < 0.05, ns = no statistical significance.

MAIT cell depletion could be also caused by an increased sequestration of the cells into the lungs, so we enumerated the total T cells and MAIT-enriched CD3^+^Vα7.2^+^ cells in the lungs of patients who succumbed to acute COVID-19. CD3^+^ cells and CD3^+^Vα7.2^+^ cell counts were calculated per 1.5 mm^2^ of lung tissue sections (Fig. 3A). CD3^+^Vα7.2^+^ cell counts in healthy controls (HC) and COVID-19 samples were normalized based on total CD3^+^ cell counts (Fig. 3B). Increased CD3^+^ cells and CD3^+^Vα7.2^+^ cells staining were observed from the COVID-19 lung samples relative to HC lungs (Fig. 3C and Supplementary Fig. 8). After normalization to total CD3^+^cells, the CD3^+^Vα7.2^+^ cell counts in COVID-19 lungs were still higher than in HC lungs, but without statistical significance, likely due to the small number of samples.

## 4 Discussion

MAIT cells are known to play important roles in the defense against viral infections. Activation of MAIT cells is observed the acute or chronic phase of HIV infection while MAITs may also play a protective role in influenza infection (Lal et al., 2020; Loh et al., 2016; Long and Hinks, 2021; Phetsouphanh et al., 2021; van Wilgenburg et al., 2016; Xia et al., 2022). Abundant evidence also shows that circulating MAITs are affected by SARS-CoV-2 infection (Deschler et al., 2021; Flament et al., 2021; Parrot et al., 2020; Youngs et al., 2021; Yu et al., 2021; Yang et al., 2021), but little is known about the mechanisms how MAIT cell lymphopenia develops in acute COVID-19. In this study, we showed that the proportion of circulating CD8^+^CD161^+^Vα7.2^+^ MAITs was significantly lower, while the remaining cells were strongly activated in the acute phase of the disease. In addition, based on the increased Ki67 expression, CD8^+^CD161^+^Vα7.2^+^ MAITs proliferated significantly in the acute disease phase, which probably at least partially contributed to their recovered frequencies during convalescence. However, the *in vitro* challenge experiments showed that neither T cells nor enriched Vα7.2^+^ cells were infected or activated by SARS-CoV-2 virus particles, suggesting that other explanations than direct virus exposure are required to understand their fate in acute COVID-19.

Human blood MAIT cells consist mainly of CD8^+^ MAIT cells (80 %) and CD4^−^CD8^−^ MAIT cells (20 %) with a small proportion of CD4^+^ MAIT cells in healthy humans (Provine and Klenerman, 2020). In agreement with previous studies (Flament et al., 2021; Parrot et al., 2020), our study identified that the proportion of circulating CD8^+^CD161^+^Vα7.2^+^ MAITs was significantly lower in acute as compared with late convalescent phase of COVID-19. Furthermore, confirming the previous reports, we observed a strong activation of CD8^+^CD161^+^Vα7.2^+^ MAITs in acute disease (Hubrack et al., 2021; Youngs et al., 2021; Yu et al., 2021). One study has shown enrichment of MAIT cells in the airways of COVID-19 patients, which could explain the corresponding decline of MAIT cells in peripheral blood (Jouan et al., 2020b). Our analysis of lung tissue from fatal COVID-19 cases suggested that CD3^+^Vα7.2^+^cells indeed accumulate in the lung during acute SARS-CoV-2 infection phase. Also, the overall T cell number was significantly higher in the infected lungs as compared to healthy controls. Infiltration of T cells and MAITs into the infected lung appears to be a significant factor behind T and MAIT cell lymphopenia in severe COVID-19.

In our study, acute samples were obtained relatively late after the symptom onset (median days 10 days, range 2–20) which could explain the lack of significant T cell lymphopenia observed in our cohort. However, CD8^+^CD161^+^Vα7.2^+^ MAITs showed significant proliferative phenotype in the acute disease phase, which could explain the rapid recovery of the diminished proportions of CD8^+^CD161^+^Vα7.2^+^ MAITs proportions at convalescence. Thus, the acute decline of circulating CD8^+^CD161^+^Vα7.2^+^ cells together with their strong activation in acute COVID-19 is in agreement with the suggested important antiviral role for MAITs against SARS-CoV-2 infection. Interestingly, ICU treated patients had lower frequency of CD8^+^CD161^+^Vα7.2^+^ MAITs during convalescence compared with hospitalized non-ICU and mild home-treated convalescent COVID-19 cases. This could be explained by the higher age of the ICU treated patients (Supplementary Table 2) as MAIT cell frequencies decline with increasing age (Chen et al., 2019). In fact, our cohort displayed a significant negative correlation between age and frequency of CD8^+^CD161^+^Vα7.2^+^ MAITs during convalescence (Supplementary Fig. 9). One possibility is that a decline in MAIT frequency and function in the elderly partly explains why age is the major risk factor for severe COVID-19.

Our study confirms that a fraction of MAIT cells express ACE2 (Liu et al., 2020) (Supplementary Fig. 2), which is recognized as the main receptor that mediates SARS-CoV-2 entrance into host cells (Tai et al., 2020). It has been extensively debated whether SARS-CoV-2 can directly infect T cells. In the case of the related Middle East Respiratory Syndrome Coronavirus (MERS-CoV), *in vitro* infection caused T cell apoptosis, which could explain lymphopenia in MERS patients (Chu et al., 2016). Shen et al. (2022) showed that SARS-CoV-2 directly infects Jurkat cell line, which consists mainly of CD4^+^ T cells, in an ACE2-independent manner. Pontelli et al. (2022), showed that T cells isolated from healthy individuals can be productively infected with the virus. The infection triggered pronounced T cell death, which could potentially also explain the T cell lymphopenia observed in COVID-19 patients. However, our study showed that *in vitro* challenge of primary T cells with purified live SARS-CoV-2 did not result in detectable infection or cell death. Interestingly, Welch et al. (2022), showed that the expression of ACE2 can be induced by activation of T cells, which suggests that activation could render T cells susceptible to infection by SARS-CoV-2. The same study also suggested that non-activated T cells could support low level infection by SARS-CoV-2 as indicated by the presence of viral RNA in virus-exposed cells. However, based on our experience input viral RNA can be detected in exposed cells for prolonged periods even without active infection/replication.

Since our study suggested that SARS-CoV-2 cannot kill, infect, or activate MAIT cells directly, other mechanistic explanations for their activation and decline in blood are needed. Alveolar macrophages are important innate immune cells for respiratory viral infections. Several studies have shown that macrophages can promote the antiviral capabilities of MAIT cells (Loh et al., 2016; van Wilgenburg et al., 2016). Interestingly, SARS-CoV-2 infected macrophages can induce MAIT activation by degranulation in an MR1-dependent manner. Moreover, infected macrophages can activate MAIT cells through the cytokine IL-18 and cause MAIT cells to switch towards a detrimental phenotype during SARS-CoV-2 infection (Flament et al., 2021). Supporting this pathway, the IL-18 concentration was higher in the acute disease phase also in our study, although we could not detect any correlation between the IL-18 levels and MAIT cell proportions in our small patient cohort. On the other hand, B cells also have the ability to affect MAIT cell functions. Upon activation, B cells express the Lectin-like transcript-1 (LLT1), which is an activating ligand for MAIT-expressed CD161 (Aldemir et al., 2005). Another study found that HLA-G expression on B cells during *Salmonella enterica* serovar Typhi infection downregulates the production of IFN-γ by MAIT cells (Salerno-Gonçalves et al., 2021). In conclusion, MAIT functions can be indirectly affected by at least macrophages and B cells during SARS-CoV-2 infection.

To our knowledge, this is the first description of MAITs in COVID-19 patients that includes the acute, early convalescent and late convalescent disease phases. MAITs constitute a relatively rare population of total circulating or tissue-resident T cells, which makes studying primary human MAITs technically challenging. We used the Vα7.2 - TCR to enrich MAITs from peripheral blood for *in vitro* challenges and to detect MAIT-like cells in the lung. Even though all MAITs are Vα7.2 - TCR positive, this population also contains non-MAIT cells which means that not all of our results are directly translatable to the MR1/5-OP-RU tetramer positive classical MAITs. Moreover, our study is limited with a relatively low number of participants divided into several subgroups as well as in the low numbers of lung tissues obtained from only fatal cases. It would be important to study the frequency and activation of MAITs in a larger cohort of older outpatients without severe COVID-19, considering how important the age of the individual as a confounder is when studying MAITs. Unfortunately our patient cohort did not allow that. Furthermore, prepandemic baseline frequencies of MAIT cells of our study participants would be required to show the full spectrum of the MAIT cell kinetics during COVID-19. Further studies determining the indirect mechanisms affecting MAIT cells by SARS-CoV-2 infection are also required.

In summary, human circulating CD8^+^CD161^+^Vα7.2^+^ MAITs are activated and their proportion has declined in circulation in patients with acute SARS-CoV-2 infection, which is not probably due to direct virus particle-mediated effects. Instead, MAITs are likely affected in an indirect way, possibly through the activation of other immune cells, such as macrophages or B cells and the cytokines they produce (Ussher et al., 2014). Taken together, our findings contribute to the understanding of the fate of MAIT cells during COVID-19 and facilitate further studies towards revealing the effects of SARS-CoV-2 infection in immune cells.

## CRediT authorship contribution statement

**Xiaobo Huang:** Conceptualization, Methodology, Validation, Formal analysis, Investigation, Data curation, Writing – original draft, Writing – review & editing, Visualization. **Jonas Kantonen:** Conceptualization, Methodology, Investigation, Resources, Writing – review & editing, Visualization. **Kirsten Nowlan:** Conceptualization, Methodology, Validation, Investigation, Writing – review & editing. **Ngoc Anh Nguyen:** Investigation, Writing – review & editing. **Suvi T. Jokiranta:** Investigation, Writing – review & editing. **Suvi Kuivanen:** Methodology, Validation, Investigation, Writing – review & editing. **Nelli Heikkilä:** Methodology, Validation, Formal analysis, Investigation, Writing – review & editing. **Shamita Mahzabin:** Conceptualization, Methodology, Investigation, Writing – review & editing. **Anu Kantele:** Conceptualization, Resources, Writing – review & editing, Supervision, Project administration, Funding acquisition. **Olli Vapalahti:** Resources, Writing – review & editing, Supervision, Project administration, Funding acquisition. **Liisa Myllykangas:** Conceptualization, Writing – review & editing, Supervision, Project administration, Funding acquisition. **Santtu Heinonen:** Investigation, Writing – review & editing. **Mikko I. Mäyränpää:** Conceptualization, Methodology, Resources, Writing – review & editing, Supervision, Project administration, Funding acquisition. **Tomas Strandin:** Conceptualization, Methodology, Formal analysis, Resources, Data curation, Writing – review & editing, Supervision, Project administration. **Eliisa Kekäläinen:** Conceptualization, Resources, Writing – review & editing, Supervision, Project administration, Funding acquisition.

## Declaration of competing interest

The authors declare that they have no known competing financial interests or personal relationships that could have appeared to influence the work reported in this paper.

## Data Availability

Data will be made available on request.

## Appendix Supplementary materials

Image, application 1
