# Supplementary material for: Mucosal-Associated Invariant T Cells are not susceptible in vitro to SARS-CoV-2 infection but accumulate into the lungs of COVID-19 patients
Source: Virus Res. 2024 Jan 13;341:199315. doi: 10.1016/j.virusres.2024.199315 (PMC10826420; doi:10.1016/j.virusres.2024.199315)

***Supplemental material***

**Supplementary Table 1.** Antibodies used in each panel

| **Antigen** | **Fluorochrome** | **Clone** | **Host** | **Cat number** | **Manufacturer** | **Titration** |
| --- | --- | --- | --- | --- | --- | --- |
| **Panel for COVID-19 kinetics** | | | | | | |
| CD8 | FITC | HIT8a | Mouse | 21810083 | Immunotools | 1:50 |
| Vα7.2 | PE | 3C10 | Mouse | 351706 | Biolegend | 1:25 |
| CD25 | PE-CF594 | M-A251 | Mouse | 562403 | BD | 1:50 |
| CXCR5 | PE-Cy7 | MU5UBEE | Mouse | 25-9185-42 | eBioscience | 1:20 |
| Foxp3 | PerCP-Cy5.5 | 236A/E7 | Mouse | 561493 | BD | 1:100 |
| CD69 | APC | FN50 | Mouse | 21620696 | Immunotools | 1:50 |
| CD3 | Alexa Fluor700 | UCHT1 | Mouse | 557943 | BD | 1:50 |
| CD4 | APC-CY7 | RPA-T4 | Mouse | 557871 | BD | 1:100 |
| CD14 | BV510 | MφP9 | Mouse | 563079 | BD | 1:50 |
| CD19 | BV510 | SJ25C1 | Mouse | 562953 | BD | 1:50 |
| CD161 | BV605 | HP-3G10 | Mouse | 339916 | Biolegend | 1:20 |
| PD1 | BV711 | EH12.1 | Mouse | 564017 | BD | 1:50 |
| Ki67 | BV786 | B56 | Mouse | 563756 | BD | 1:50 |
| **Panel for *in vitro* viral challenge** | | | | | | |
| CD8 | FITC | HIT8a | Mouse | 21810083 | Immunotools | 1:50 |
| HLA-DR | Alexa Fluor700 | G46-6 | Mouse | 560743 | BD | 1:50 |
| MR1 | APC | NIH tetramer core facility | | | | 1:2500 |
| CD4 | APC-CY7 | RPA-T4 | Mouse | 557871 | BD | 1:100 |
| CD3 | BV786 | SK7 | Mouse | 563800 | BD | 1:50 |
| **Antibodies for ACE2 flow cytometry** | | | | | | |
| ACE2 | - | - | Goat | AF933-SP | R&D System | 1:10 |
| Goat IgG | Alexa Fluor 488 | - | Rabbit | - | Invitrogen | 1:250 |

**Supplementary Table 2.** Clinical information of COVID-19 patients in convalescent disease phase

| Characteristic | | ICU treated patients | Hospitalised patients | Home treated patients |
| --- | --- | --- | --- | --- |
| n (%) | Total | 10 (100) | 10 (100) | 14 (100) |
|  | Early convalescent | 3 (30) | 2 (20) | 7 (50) |
|  | Late convalescent | 7 (70) | 8 (80) | 7 (50) |
| Age, years (median) | | 59** | 56 | 43 |
| Age, years IQR | 25^th^ | 52.3 | 35.5 | 32.8 |
|  | 75^th^ | 64.5 | 63.25 | 56.0 |
| Female (%) | | 5 (50) | 6 (60) | 8 (57) |
| Male (%) | | 5 (50) | 4 (40) | 6 (42) |
| DSO (median) | | 99 | 273 | 27 |
| DSO range | | 23 - 112 | 26 - 417 | 22 - 81 |
| DSO IQR | 25^th^ | 24 | 91 | 25 |
|  | 75^th^ | 107 | 395 | 36 |

*IQR: Interquartile range; DSO: Days from symptom onset before sampling; **P < 0.01.*

**Supplementary Figure 1.** MAITs, T cell purity after cell isolation and overlap between CD8^+^ MR1/5-OP-RU^+^ MAITs and CD8^+^ Vα7.2^+^ CD161^+^ MAITs... (A) MAIT cells purity as measured by MR1/5-OP-RU tetramer positivity after MicroBeads isolation. (B) T cells purity after Pan T Cell Isolation as measured as CD3-positivity. (C) Overlap between CD8^+^ MR1/5-OP-RU^+^ MAITs and CD8^+^ Vα7.2^+^ CD161^+^ MAITs in a subset of our COVID-19 patient samples (n=38). Data were shown as median with range.


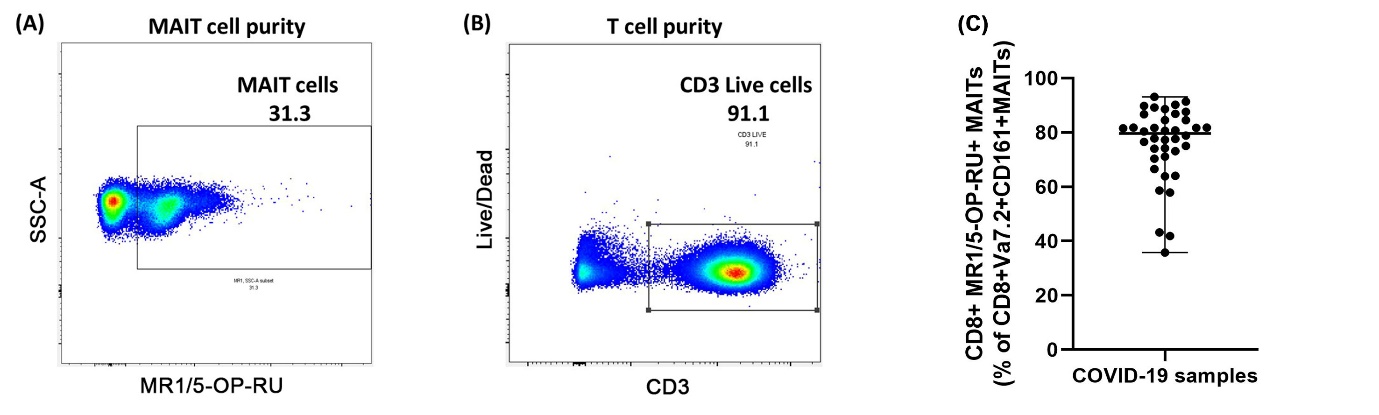


**Supplementary Figure 2.** ACE2 expression on CD3^+^MR1/5-OP-RU^+^ cells. (A) Unstained sample, (B) only ACE2 primary antibody stained sample, (C) only Goat IgG - Alexa Fluor 488 secondary antibody stained sample, (D) both primary (ACE2) and secondary (Alexa Fluor 488) antibody stained sample, parent gate is live lymphocytes. ACE2 expression on CD3^+^ MR1/5-OP-RU^+^ cells of (E) donor 1, (F) donor 2, (G) donor 3. (H) Histograms of ACE2 expression on CD3^+^ MR1/5-OP-RU^+^ cells with 3 donors.

**
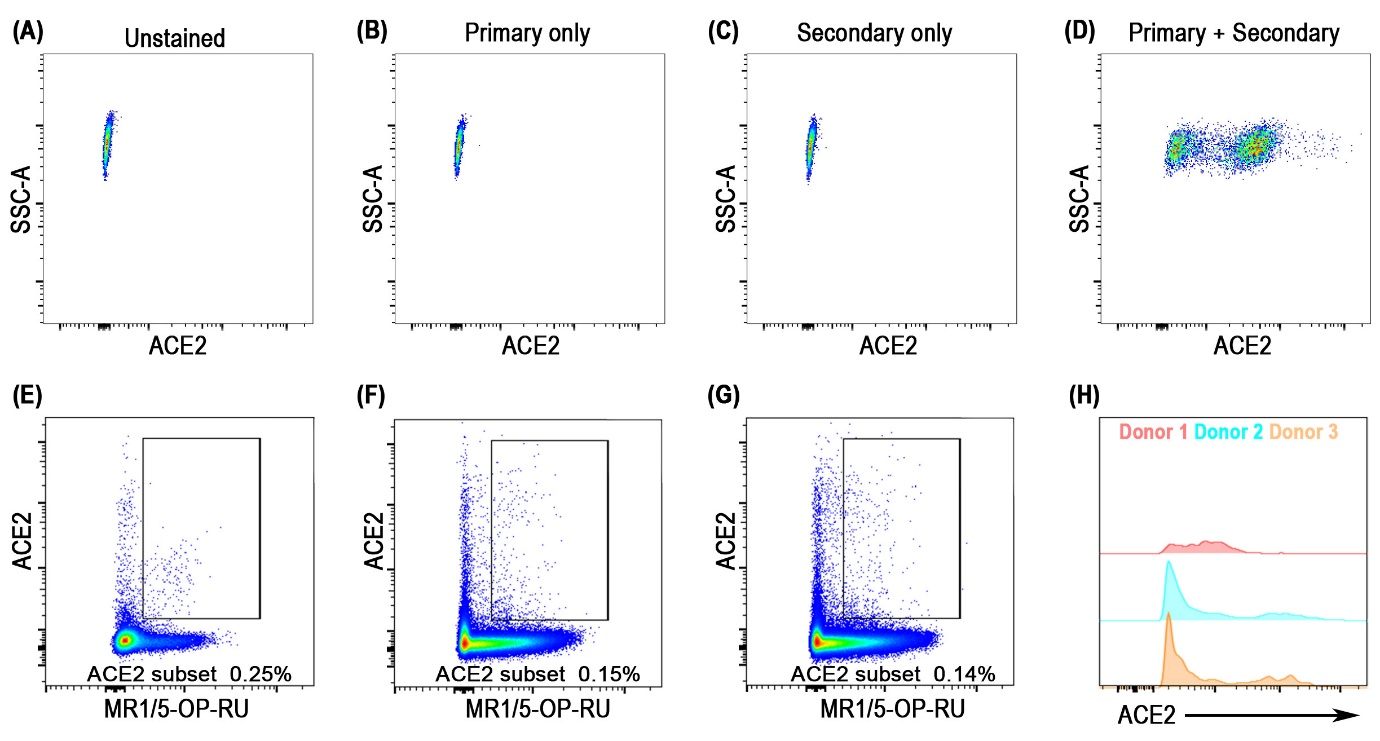
**

**Supplementary Figure 3**. Pseudo-color immunofluorescence images representative of healthy control and COVID-19 lung tissue sections at 20 x magnification. No primary antibodies were used to highlight the level of autofluorescence present in lung tissue.

**
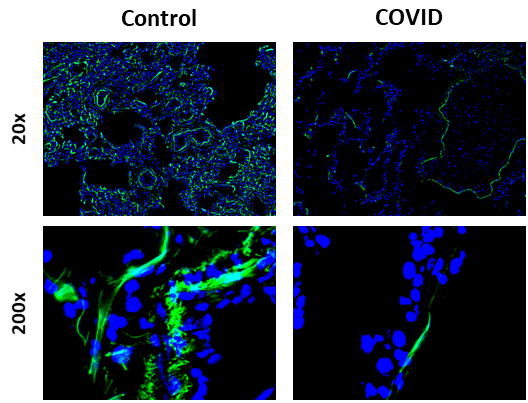
**

**Supplementary Figure 4.** Gating strategy


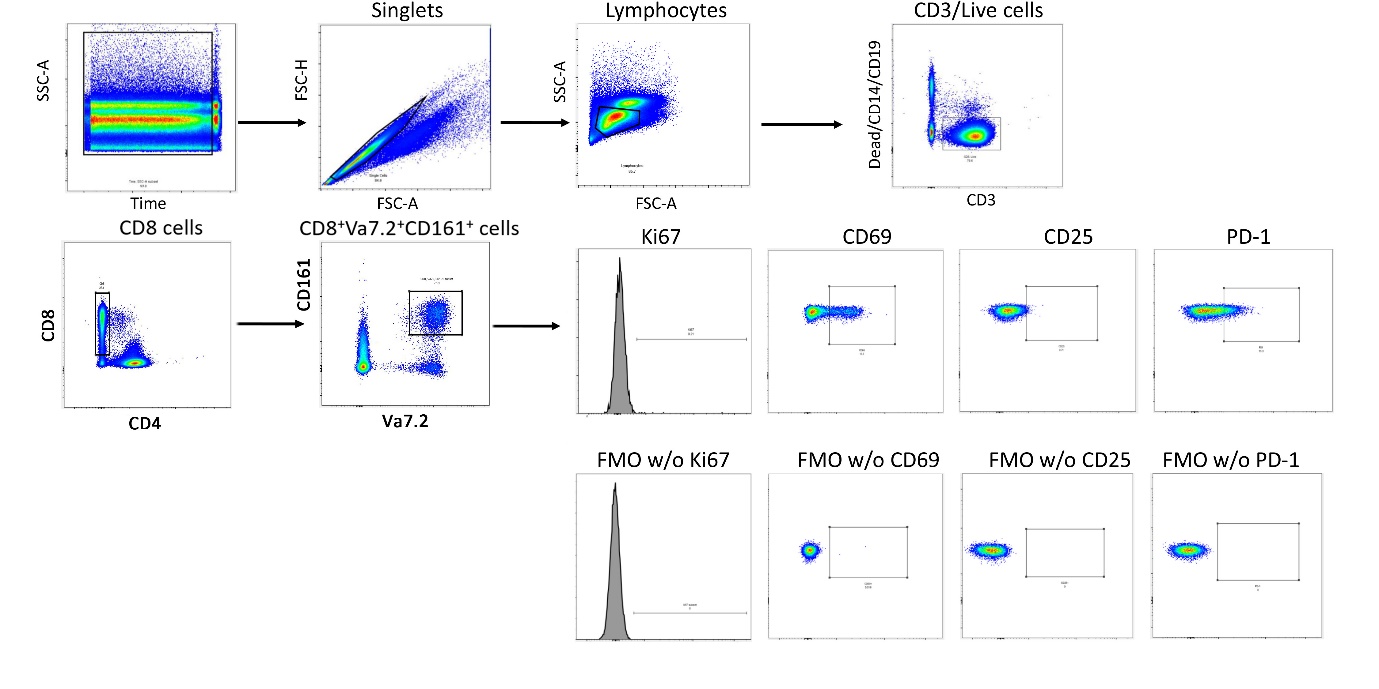


**Supplementary Figure 5.** Unpaired acute patient samples analysis among ICU-treated and hospitalised patients. (A) The proportion of CD8^+^ Vα7.2^+^ CD161^+^ MAITs of CD8 cells. (B) The proportion of Ki67^+^cells of CD8^+^ Vα7.2^+^ CD161^+^ MAITs. Data were shown as means ± SD. Wilcoxon and Mann-Whitney U tests were used to test for statistical significance. No statistical significance was observed.

**
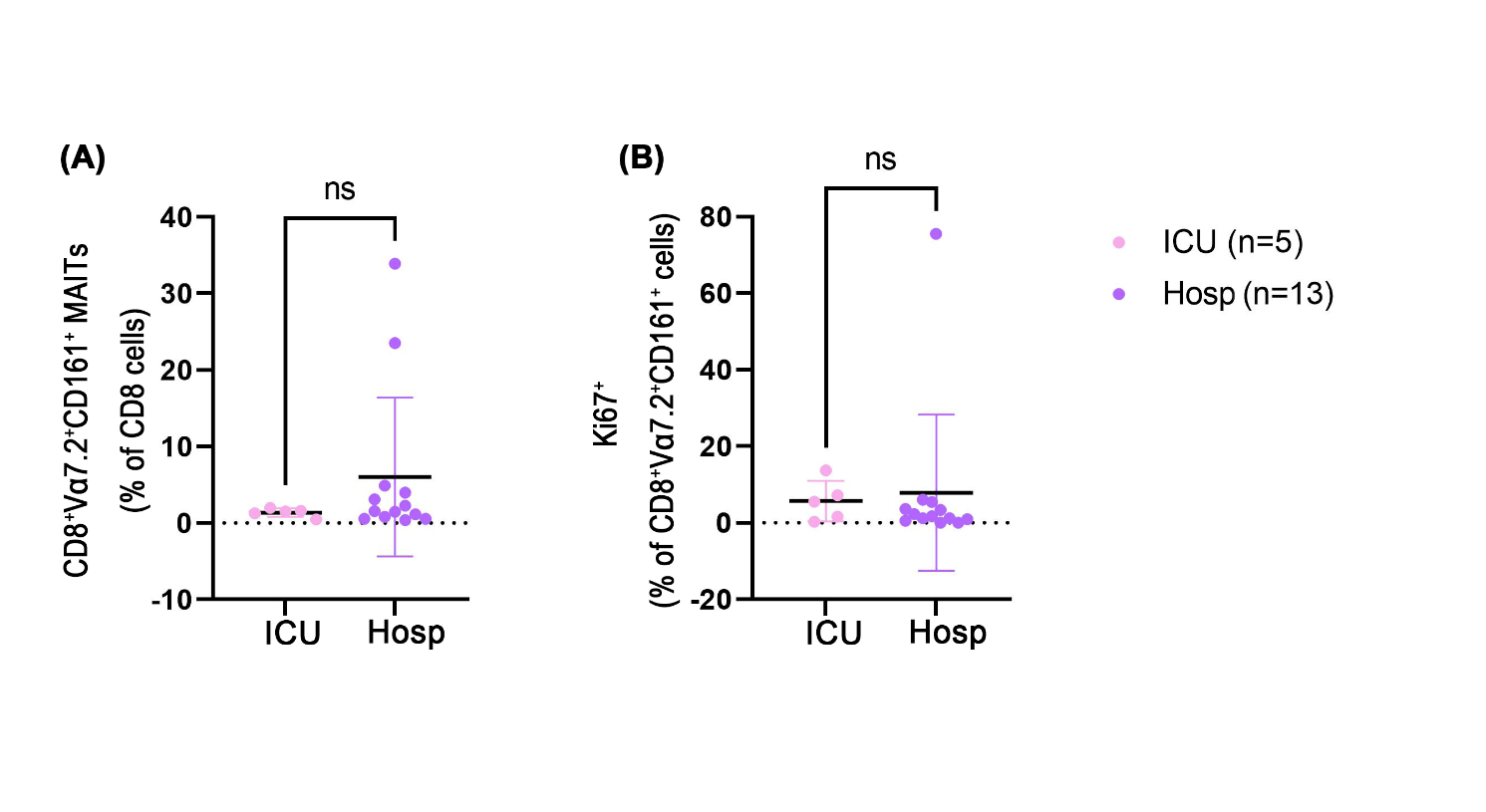
**

**Supplementary Figure 6.** The viability of enriched MAIT cells after *in vitro* challenge with live SARS-CoV-2 (MOI 1) for 48h. Kruskal-Wallis one-way ANOVA was used to test for statistical significance. No statistical significance was observed.

**
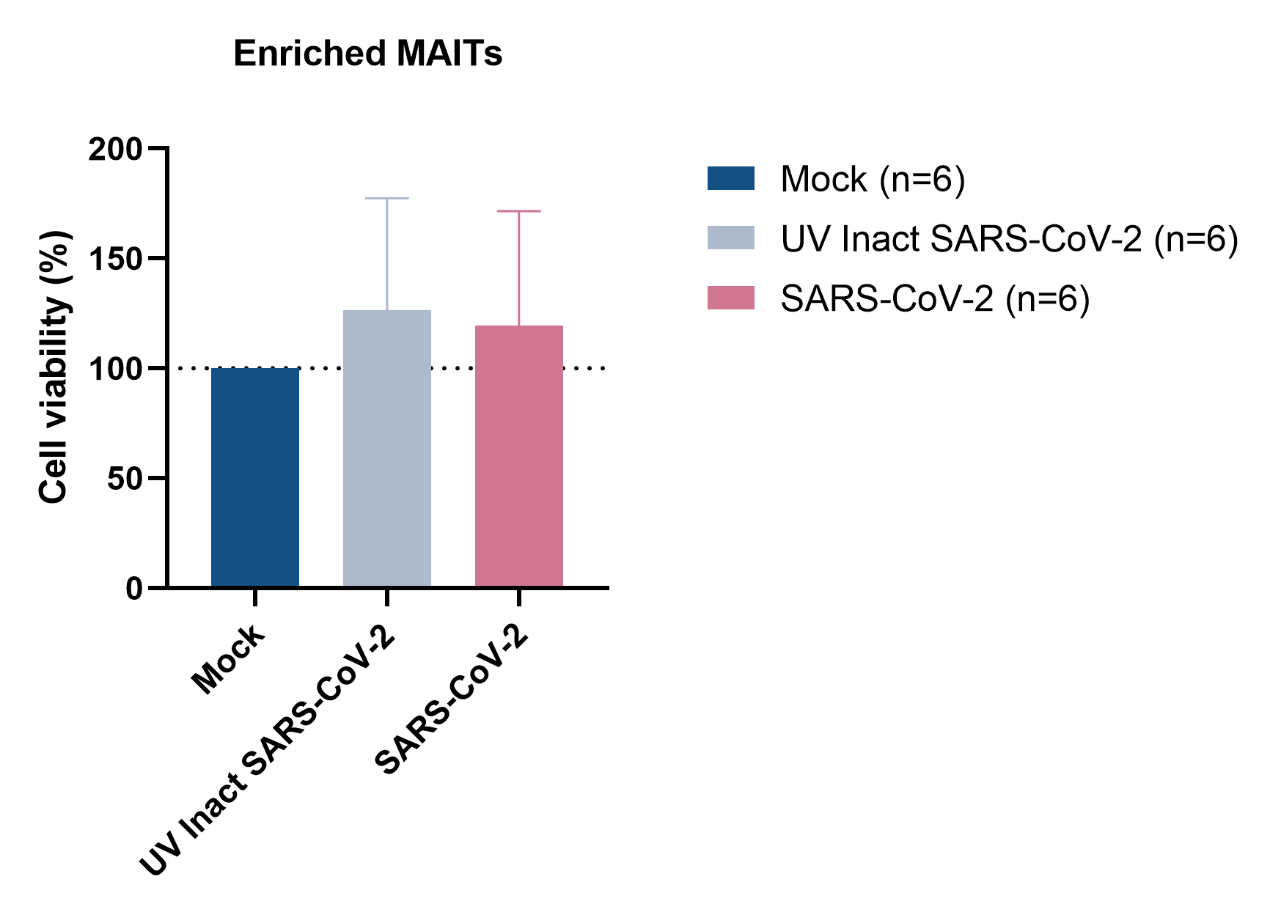
**

**Supplementary Figure 7.** Peripheral IL-18 concentration. (A) Peripheral IL-18 concentration in unpaired acute (n=8) and late convalescent (n=9) phases. (B) Peripheral IL-18 concentration in paired acute (n=6) and late convalescent (n=6) phases. A, Acute. LC, Late convalescent. Data were shown as mean ± SD. Mann-Whitney U tests were used when comparing between two unpaired groups. Wilcoxon signed rank tests were used with paired group comparison. **P* < 0.05, ***P* < 0.01, ****P* < 0.001.

**
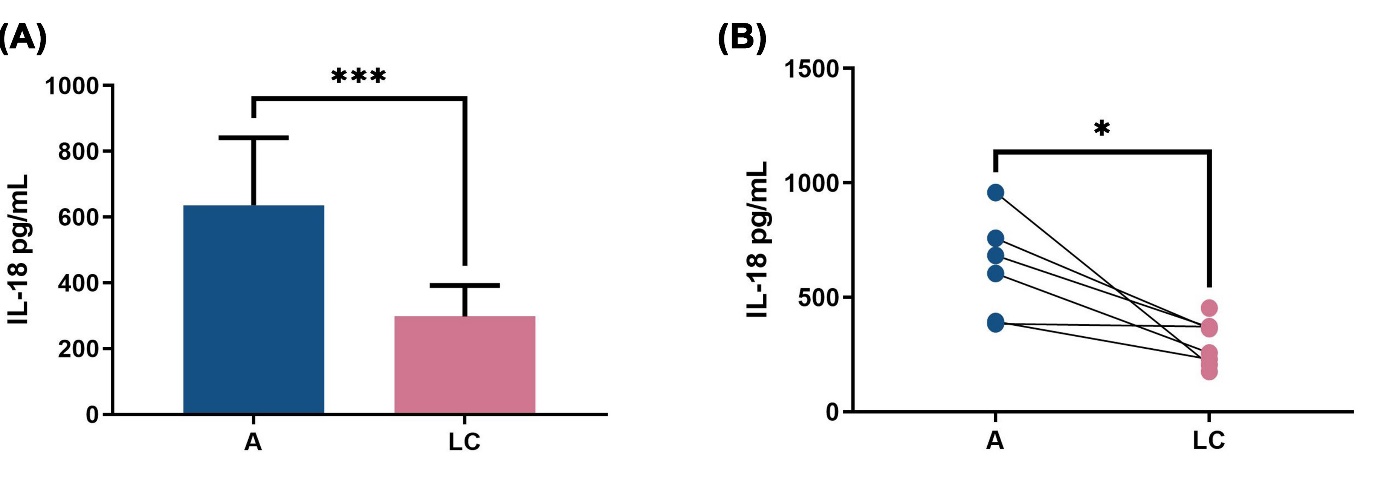
**

**Supplementary Figure 8.** Panel of pseudo-color immunofluorescence images representative of healthy control (HC) and COVID-19 lung tissue sections at 63 x magnification all channels merged and representative annotated areas at 20 x magnification. Autofluorescent fibres are seen in the green channel. Increased CD3^+^ cells (green cytoplasmic staining) and CD3^+^Vα7.2^+^ cells (green and white cytoplasmic staining) were observed from the COVID-19 lung samples relative to normal lungs.
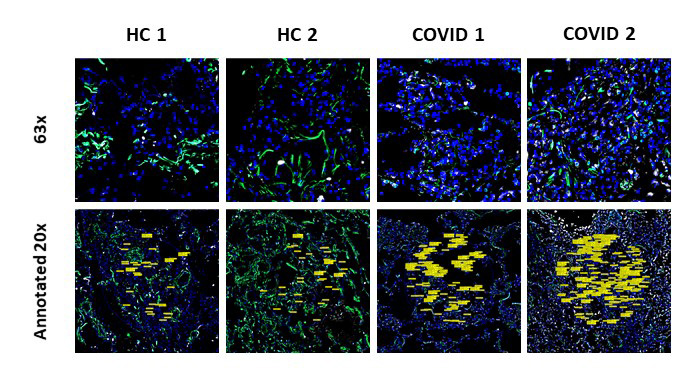


**Supplementary Figure 9.** Correlation matrix between demographic details and CD8^+^Vα7.2^+^CD161^+^ MAITs frequencies of all convalescent samples. Patient age and treatment have a significant positive correlation (r = 0.4508, *P*=0.007). Patient age and the proportion of CD8^+^ Vα7.2^+^ CD161^+^ MAITs of CD8 cells have a negative correlation (r = -0.4550, *P*=0.007). Treatment was code as home-treated = 0, hospitalised = 1, ICU = 2. Gender was coded as female = 0, male = 1. X indicates no significant correlation. *P* values were calculated using the Spearman’s correlation test and correlations with *P* values ≥ 0.05 are crossed over.


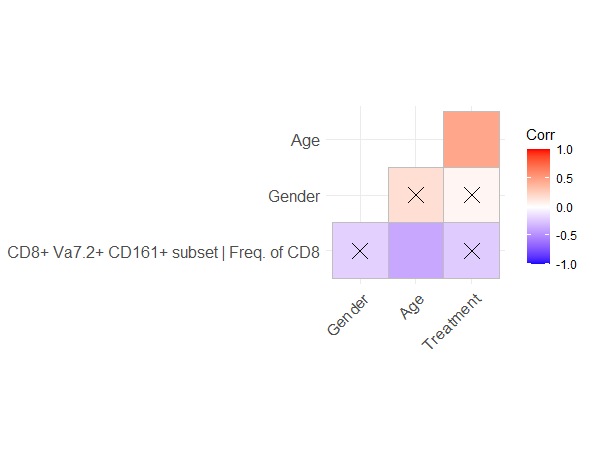

Supplement: Supplementary file 1 [file mmc1.docx]
